# Advances of the MAPK pathway in the treatment of spinal cord injury

**DOI:** 10.1111/cns.14807

**Published:** 2024-06-17

**Authors:** Shixue Huang, Yinuo Zhang, Haoming Shu, Wei Liu, Xin Zhou, Xuhui Zhou

**Affiliations:** ^1^ Department of Orthopedics, Changzheng Hospital Second Affiliated Hospital of Naval Medical University Shanghai China; ^2^ Translational Research Centre of Orthopedics, Shanghai General Hospital Shanghai Jiao Tong University School of Medicine Shanghai China

**Keywords:** axonal regeneration, inflammation, MAPK signaling pathway, spinal cord injury, therapeutic approaches

## Abstract

Spinal cord injury (SCI) represents a complex pathology within the central nervous system (CNS), leading to severe sensory and motor impairments. It activates various signaling pathways, notably the mitogen‐activated protein kinase (MAPK) pathway. Present treatment approaches primarily focus on symptomatic relief, lacking efficacy in addressing the underlying pathophysiological mechanisms. Emerging research underscores the significance of the MAPK pathway in neuronal differentiation, growth, survival, axonal regeneration, and inflammatory responses post‐SCI. Modulating this pathway post‐injury has shown promise in attenuating inflammation, minimizing apoptosis, alleviating neuropathic pain, and fostering neural regeneration. Given its pivotal role, the MAPK pathway emerges as a potential therapeutic target in SCI management. This review synthesizes current knowledge on SCI pathology, delineates the MAPK pathway's characteristics, and explores its dual roles in SCI pathology and therapeutic interventions. Furthermore, it addresses the existing challenges in MAPK research in the context of SCI, proposing solutions to overcome these hurdles. Our aim is to offer a comprehensive reference for future research on the MAPK pathway and SCI, laying the groundwork for targeted therapeutic strategies.

## Graphical abstract

The activation of MAPK pathway promotes neuroinflammation, neuropathic pain, and neuronal apoptosis. Molecular biology experiments targeting this pathway primarily revolve around drug interventions, stem cell transplantation, and other aspects. Additionally, clinical trials related to this area are progressing vigorously.

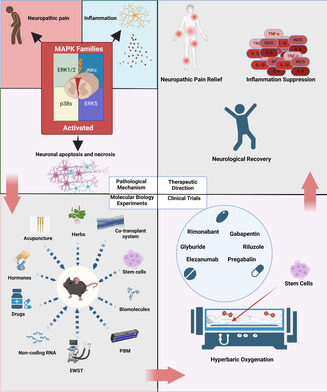

## 1 INTRODUCTION

Spinal cord injury (SCI) is characterized by high morbidity, disability, substantial economic impact and a tendency to affect individuals at a younger age.^1^ These injuries, often resulting from high‐impact events such as traffic accidents, falls, and acts of violence, can also occur due to infections, tumors, degenerative spinal conditions, ischemia–reperfusion injuries, and vascular diseases.^2^ An epidemiological study from 1990 to 2019 indicates a consistent increase in SCI prevalence, with an estimated 200 million people globally affected in 2019, underscoring the profound physical and social implications of this condition.^3^ Over the last 30 years, the global number of cases increased from 236 to 1298 per million population. The global incidence of SCI is estimated to be 250,000–500,000 cases per year.^4^ On a global scale, the incidence of SCI is relatively high and shows a consistent upward trend.^5^ In North America, the National Spinal Cord Injury Statistical Center estimates 12,500 annual new cases of SCI.^6^ Research indicates a rapid increase in the incidence of traumatic SCI in China from 2009 to 2018. Estimates reveal that in 2009, there were approximately 59,685 cases, a number that escalated to around 92,841 cases by 2018, indicating a rapid and persistent rise in the incidence rate.^7^ SCI is broadly categorized into primary and secondary injuries. Primary injury refers to the immediate tissue and cellular damage inflicted by external forces, while secondary injury encompasses a cascade of pathological changes stemming from the initial trauma, which include ionic imbalance, free radical generation, lipid peroxidation, inflammation, cell death, demyelination, axonal degeneration, excitatory neurotransmitter buildup and glial scar formation.^8^ Given that primary injuries are often irreversible, clinical interventions primarily aim at mitigating the pathological progression of secondary injuries, though effective treatment strategies remain limited.^9^

After SCI, several signaling pathways are activated, including the transforming growth factor (TGF)‐β signaling pathway, phosphatidylinositol 3‐kinase /protein kinase B (PI3K/Akt) signaling pathway, nuclear factor kappa‐B (NF‐κB) signaling pathway and MAPK pathways. The MAPK pathway, in particular, is critical in regulating cellular processes such as proliferation, differentiation, and apoptosis. It comprises four major groups: extracellular signal‐regulated kinases 1/2(ERK1/2), the p38 family (α, β, γ, and δ), the c‐Jun N‐terminal kinases families (JNK1, JNK2, and JNK3), and ERK5.^10^ Recent research has increasingly focused on the MAPK pathway due to its pivotal role in inflammatory diseases, cancer, stem cell biology, and targeted drug therapies.^11^, ^12^, ^13^ This article reviews the pathological process of SCI, the regulation of the MAPK signaling pathway in SCI, and the advances in research in SCI treatment.

## 2 LITERATURE SEARCH STRATEGY

To conduct our analysis, we undertook a comprehensive literature review utilizing three major databases: PubMed, Web of Science, and EMBASE. Our search parameters focused on key terms including “spinal cord injury,” “MAPK signaling pathway,” “inflammation,” and “neuroprotection.” Our group meticulously examined articles pertaining to the pathophysiology of SCI, the immune response subsequent to SCI, and the involvement of the MAPK signaling pathway post‐SCI. Rigorous filtering procedures were implemented to exclude outdated and duplicate studies, thereby ensuring the precision and pertinence of the findings.

## 3 PATHOLOGY OF SCI

SCI is classified into two distinct phases based on the progression and pathological changes within the spinal cord following an injury: primary and secondary injury phases (Figure S1). Primary injury, typically arising from mechanical factors like impact, compression, transection, and laceration, inflicts direct damage to neuronal and vascular tissues. This results in acute cellular dysfunction and cell death.^14^, ^15^, ^16^ Acute SCI predominantly exhibits complete functional impairment, though research indicates that a small proportion of cases show anatomically partial or incomplete damage, preserving some axonal connections.^17^ Timely intervention at the injury site is crucial for anatomical reconstruction and neurological recovery. The secondary injury phase, ensuing after the primary injury, can extend for hours, days, or even weeks. It involves processes distinct from those of primary injury, such as neuroinflammation, ischemia, free radical production, lipid peroxidation, disruption of the blood‐CNS barrier, edema, protease release, and neuronal excitotoxicity. These processes exacerbate acute cellular dysfunction and cell death.^18^, ^19^

Secondary injury can be further segmented into acute, subacute, and chronic phases. The acute phase, following the primary injury, is marked by vascular damage, ionic imbalance, increased intracellular calcium, free radical generation, lipid peroxidation, inflammatory responses, edema, and cellular necrosis.^14^ If these pathophysiological changes persist, the injury progresses to the subacute phase, characterized by neuronal apoptosis, Wallerian degeneration, axonal demyelination, remodeling and glial scar formation.^14^ The chronic phase, succeeding the subacute phase, involves capsular cavity formation, axonal degeneration, and maturation of glial scars.^20^ While these stages offer a framework for understanding SCI pathology, the real situation is more complex. Multiple factors co‐occur, leading to intricate pathophysiological shifts and challenging the treatment of SCI.

The overlapping and interdependent pathological changes at each stage catalyze a cascade of reactions, critically shaping the progression and regression of SCI. Identifying targets to modulate the secondary injury's pathological process, particularly through early intervention and halting lesion progression, is vital for neurological recovery and improving clinical outcomes post‐SCI.

## 4 SIGNALING PATHWAY POST‐SCI

SCI triggers complex cellular responses, including alterations in various signaling pathways, which significantly influence the pathophysiological processes post‐injury. Among the key signaling pathways affected post‐SCI are the MAPK pathway, the NF‐κB pathway, and the PI3K/Akt pathway. Activation of the MAPK pathway, particularly the p38 MAPK and JNK pathways, has been implicated in neuroinflammation, apoptosis, and glial scar formation post‐SCI.^21^ The NF‐κB pathway plays a crucial role in the transcriptional regulation of proinflammatory cytokines and cell survival genes post‐SCI, contributing to the inflammatory response and tissue damage.^22^ Conversely, activation of the PI3K/Akt pathway has been associated with neuroprotection, promoting cell survival, axonal regeneration, and functional recovery post‐SCI.^23^ Understanding the dynamic changes in these signaling pathways post‐SCI is essential for developing targeted therapeutic strategies to mitigate secondary injury and enhance neurological recovery.

As previously described, the pathologic process following SCI is complex both temporally and spatially. This process involves multiple systems, including the nervous and immune systems. Physiological processes such as necrosis and apoptosis of nerve cells, synthesis and release of cytokines form a spatial and temporal network that varies in the state of different species and individual organisms, yet still has a certain pattern. Therefore, it is of practical significance to investigate the activation of signaling pathways after SCI.

## 5 THE MAPK SIGNALING PATHWAY

Recently, the MAPK signaling pathway has received more attention in neurological diseases, especially neurodegenerative diseases, such as Parkinson's disease^24^ and Alzheimer's disease.^25^ The pathologic changes in SCI have similar mechanisms to those in neurodegenerative diseases. Thus, the MAPK signaling pathway holds significant research implications for both the understanding of SCI pathogenesis and the development of therapeutic interventions.

**FIGURE 1:**
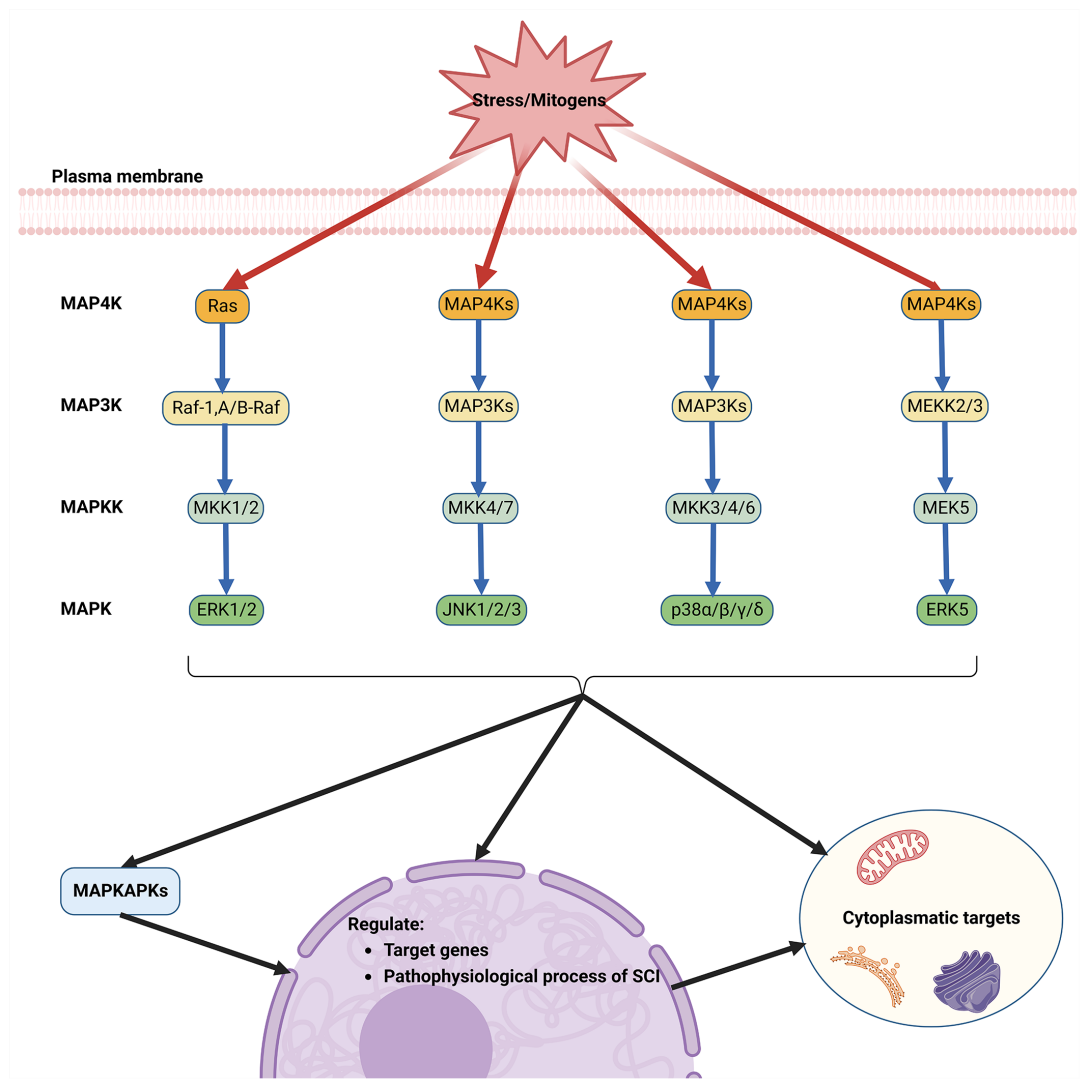
MAPK signaling pathway cascade. Depicts the signal transduction through the MAPK pathway, activated by stressors, mitogens, and other factors. It illustrates four classical pathways: p38, JNK, ERK1/2, and ERK5, each playing a role in regulating nuclear genes, cell metabolism, cell cycle, and other important cellular processes. The cascade of each pathway is detailed through five major levels: MAP4K, MAP3K, MAPKK, MAPK, and MAPKAPK.

Once SCI happens, a large number of stimuli are pressured into the damaged region, producing a series of biological effects. The MAPK signaling pathway, responsive to a myriad of extracellular stimuli, stands as a crucial and universally conserved intra‐ and extracellular signaling conduit in plants,^26^ fungi, and animals.^27^, ^28^ This multifaceted pathway plays a pivotal role in regulating a diverse array of cellular programs, such as embryogenesis, cell growth, proliferation, differentiation, stress response, immune reaction, and apoptosis.^10^, ^29^ These processes are modulated depending on the nature of the extracellular cues, environmental context, and the cellular metabolic state. The architecture of each MAPK cascade typically features three core kinases: MAP3K, MAPKK, and MAPK, which are frequently accompanied by upstream MAP4K and downstream MAPKAPK components.^30^ MAPKs are classified into two major groups based on their phosphorylation and activation by MAPKK/MEK family members. The classical MAPK group encompasses extracellular signal‐regulated kinases 1 and 2 (ERK1/2), the p38 MAPK family (α, β, γ, and δ variants), the c‐Jun amino‐terminal kinases (JNKs: JNK1, JNK2 and JNK3) and ERK5. Additionally, other kinases bearing sequence resemblance to MAPK cascade components have been identified, such as ERK3/ERK4, NLK, and ERK7. These kinases exhibit stark contrasts to classical MAPKs in both structural makeup and activation patterns, suggesting they diverge from the conventional components of the MAPK response (Figure 1).

The ERK1/2 cascade, recognized as the archetypal MAPK cascade response, was the first to be delineated and is considered the most quintessential.^31^ It is activated by a spectrum of stimuli including growth factors, cytokines, viruses, G protein‐coupled receptor ligands, and oncogenes. This pathway involves a sequential activation of the upstream activator protein Ras, followed by Raf, MEK, and culminating in ERK. The initiation of ERK1/2 activation typically begins with the activation of RAS by small GTPases at the plasma membrane, leading to the recruitment and activation of MAP3K. This event is followed by the phosphorylation of two serine residues in the activation loop of MAPKK (MEK1/2), which then transmits the signal to ERK1 and ERK2. These kinases undergo phosphorylation in their Thr‐Glu‐Tyr activation loop structure, facilitating the relay of signals to MAPKAPK components and other structures in the cytoplasm, nucleus, and various organelles. Upon receiving stimulus signals, ERK1/2 and MAPKAPK components phosphorylate a multitude of substrates across different cellular locations, orchestrating a range of processes including proliferation, differentiation, morphological determination, neuronal survival and plasticity, stress response, and apoptosis.^32^, ^33^

The JNK signaling pathway, initially regarded as a regulator of the transcription factor c‐Jun and a mediator of intra‐ and extracellular stress, is also known as the stress‐activated protein kinases (SAPKs) signaling pathway.^34^ Similar to other MAPK signaling routes, it is activated by receptors in response to mitogens and various non‐stress‐related stimuli.^35^ The signaling cascade typically involves the conveyance of signals to small GTPases (e.g., CDC42 and Rac1), which either directly activate MAP3Ks or do so indirectly via MAP4Ks.^36^ This leads to the subsequent phosphorylation of MAPKK (MKK4 and MKK7), activating JNKs and downstream MAPKAPKs. These kinases then phosphorylate a plethora of substances, predominantly in the nucleus, further regulating gene transcription and mediating cellular functions such as apoptosis, immune responses, neuronal activity, insulin signaling and more.^30^ The JNK signaling pathway plays a critical role in various neural processes, including neuronal cell injury, neuroinflammation, axonal growth, regeneration, degeneration, and the formation of glial scars.^37^, ^38^, ^39^

The p38 signaling pathway, another branch of the SAPKs, shares numerous elements with the stress‐induced JNK pathways.^40^ When receptors are stimulated or environmental factors induce stress, the p38 pathway transmits signals through a series of proteins, including small GTPases, MAP4Ks, and MAP3Ks. The MAPKK components of this pathway, primarily MKK3 and MKK6 (and occasionally MKK4), undergo phosphorylation and activation.^41^ Subsequently, the four isoforms of p38 (α, β, γ, δ) are phosphorylated and activated. These activated kinases relay signals to target molecules, which are instrumental in inducing and regulating various cellular processes such as inflammatory responses, apoptosis, cell cycle changes, cell growth, development, cellular differentiation, senescence and tumor suppression.^42^ The p38 pathway is notably activated by stress signals and plays an essential role in immune response, as well as in regulating cell survival and differentiation.^43^

ERK5 was independently identified in 1995 by two research teams. It was initially linked to MAPK activation in response to oxidative and osmotic stress.^44^ Current knowledge posits that ERK5 is activated by extracellular stimuli like platelet‐derived growth factor (PDGF), vascular endothelial growth factor (VEGF), fibroblast growth factor (FGF) and epidermal growth factor (EGF), as well as by physiological and pathological conditions such as laminar shear, ischemia and hypoxia.^45^ The ERK5 signaling pathway follows a tertiary kinase cascade, where various extracellular stimuli activate MEKK2/3, which then activates MEK5, leading to the activation of ERK5.^46^ ERK5 can act as a transcription factor, mediating transcription processes by binding to DNA through its non‐catalytic region.^47^ Research has demonstrated that ERK5 activation is closely associated with cell proliferation, angiogenesis, immune response and stress response,^30^ playing a crucial role in numerous fields including cancer,^48^ diabetic retinopathy,^49^ cardiovascular disease,^50^, ^51^ and others.

In summary, ERK1/2, JNKs, p38s, and ERK5 are indispensable intermediaries in the MAPK signaling pathway, crucial in the regulation of essential physiological processes like cell growth, development, proliferation, differentiation, apoptosis, inflammatory cell activation, and inflammatory factor release. The abundance of stressors in SCI can lead to aberrant activation of the MAPK signaling pathway, negatively impacting the treatment and prognosis of SCI. Consequently, modulating the MAPK signaling pathway presents a promising avenue for SCI treatment.

## 6 ROLES OF THE MAPK SIGNALING PATHWAY IN SCI

The intricate pathological changes in secondary SCI involve numerous mechanisms and signaling pathways that influence the progression and prognosis of the condition. Each member of the MAPK family, pivotal in local inflammation, assumes a critical role in this stage of SCI. Notably, inhibition of p38 effectively suppresses the release of inflammatory mediators and attenuates neuronal apoptosis, thereby facilitating neurological recovery.^52^ Similarly, inhibition of ERK1/2 has demonstrated comparable efficacy to p38 inhibition.^53^ Moreover, inhibition of JNKs prevents retrograde axonal degeneration and preserves corticospinal tract (CST) axonal integrity post‐SCI, consequently promoting motor function.^54^ The MAPK signaling pathway, known for its extensive involvement in various cellular physiological and pathological activities, is the focal point of this review. It examines the MAPK pathway's regulatory role in neuronal survival, oligodendrocyte apoptosis, inflammatory response, and axonal regeneration post‐SCI.

### 6.1 Regulation of neuronal survival and oligodendrocyte apoptosis

Neuronal destruction, a direct and severe consequence of SCI, encompasses acute neuronal cell dysfunction, apoptosis, necrosis, and disintegration. This is initially due to the primary direct impact and subsequently aggravated by inflammatory cell infiltration and inflammatory factors during secondary injury.

The induction of neuronal apoptosis post‐SCI, closely associated with apoptosis signaling‐mediated kinase 1 (ASK1), has been shown to be significantly influenced by the p38 and JNK signaling pathways.^55^ Contrasting with these pathways, ERK1/2 is involved in neuroprotective mechanisms, regulating the pro‐ and anti‐apoptotic members of the Bcl‐2 family, thereby exerting a protective influence on neurons.^56^ Neuronal damage is often accompanied by harm to oligodendrocytes, the primary constituents of the myelin sheath. Studies, such as those by Li et al.,^57^ have demonstrated that oligodendrocyte apoptosis is differentially regulated by JNK3 and proteins that interact with mitotic kinases.

### 6.2 Promotion of the inflammatory response

The inflammatory response, a critical pathological change in secondary SCI, can lead to axonal degeneration, neuronal and oligodendrocyte death, and glial scar formation, significantly impairing neuronal function.^58^ These changes expand the lesions and exacerbate their severity, directly impacting patient prognosis.

During the secondary phase of SCI, the production of inflammatory cytokines and chemokines results in extensive infiltration of immune cells, including microglia, peripherally derived macrophages (PDMs), and neutrophils. These cells continue to produce further inflammatory mediators. While these infiltrating leukocytes and activated glial cells exacerbate tissue damage through proinflammatory cytokines/chemokines, proteases, and reactive oxygen intermediates, they also exhibit beneficial aspects in some scenarios.^59^ Inflammation post‐SCI is complex, involving multiple cell types and various inflammatory cytokines, with IL‐6, TNF‐α and IL‐1β playing crucial roles in secondary pathological changes.^60^ Upon stress stimuli, innate glial cells in the spinal cord activate the MAPK signaling pathway, leading to glial cell activation and the release of inflammatory cytokines. Among the MAPK family members, p38 is a central regulator of macrophages and dendritic cells post‐SCI, modulating cytokines like inducible nitric oxide synthase (iNOS), IL‐6 and TNF‐α and thereby influencing the inflammatory response.^59^ Numerous studies have highlighted the significant role of microglia in the post‐SCI inflammatory response, with p38,^61^ ERK1/2,^62^ ERK5,^63^ and JNK^64^ implicated in the polarization and activation of glial cells at the SCI site.

### 6.3 Inhibition of axonal regeneration

Axonal rupture post‐SCI is a key factor disrupting nerve conduction, making axonal regeneration crucial for the recovery of motor and neurological functions. The proximal ends of severed axons form retractile bulbs that persist for months, further losing neuronal connectivity and hindering axonal regeneration, which limits functional recovery post‐SCI.^54^

While JNKs are known for their axonal degenerative activity in SCI, numerous reports suggest their important role in axon guidance and neurite growth. Yoshimura et al.^54^ observed that the expression of activated JNK increased in injured spinal cord tissue on days 1 and 3 after injury and remained increased on days 5 and 7 by Western blot (WB) assay of activated JNK at the site of SCI. Immunohistochemistry and double‐labeling experiments revealed that activated JNK co‐localized with SMI‐31 (phosphorylated neurofilaments) in the white matter near the lesion site at 1, 3, 7, and 14 days after SCI, indicating JNK activation in the axons. Significantly, axonal retraction post‐SCI was effectively blocked by the JNK inhibitor SP600125, underscoring that JNK induced axonal retraction and inhibited axonal regeneration post‐SCI.

## 7 ROLES OF MAPK IN THE TREATMENT OF SCI

**FIGURE 2:**
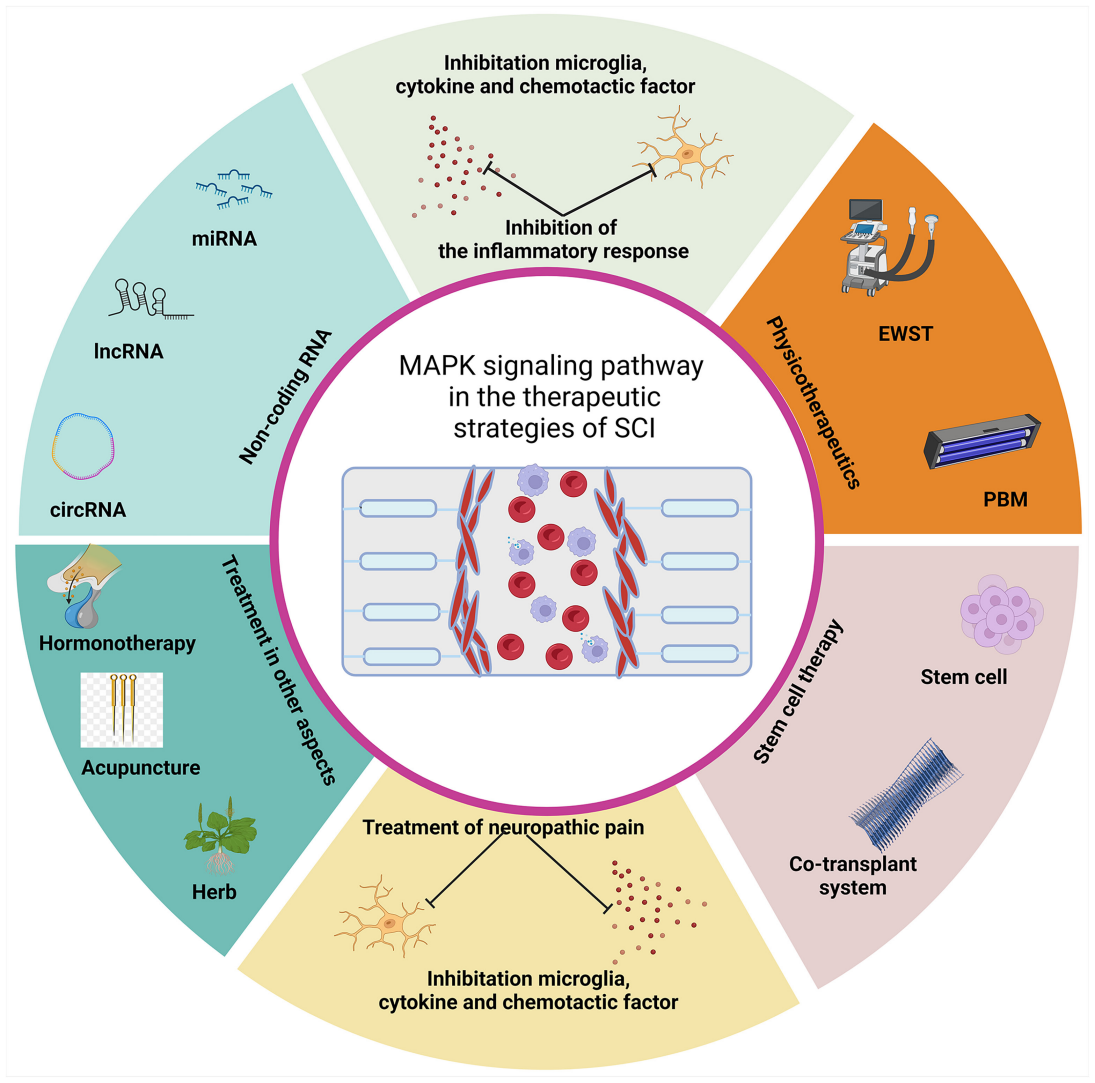
MAPK pathway‐related treatments in SCI. Outlines various therapeutic strategies for SCI via the MAPK signaling pathway. These include alleviating neuropathic pain, inhibiting inflammatory responses, applying stem cell therapy, utilizing non‐coding RNA, and employing physicotherapeutics.

The treatment strategies for SCI, a condition marked by its irreversibility and complexity, are geared toward symptomatic relief and the restoration of neurological functions. Emerging studies have highlighted the role of the MAPK signaling pathway in SCI treatment, focusing on neuropathic pain alleviation, reduction of inflammatory responses, stem cell therapy, and non‐coding RNA‐related therapies (Figure 2, Table 1).

**TABLE 1:** Treatment of SCI via the MAPK signaling pathway.

Regulation modes/regulators	Mechanism	Functions	References
Regulators in neuropathic pain			
SB203580 (p38 MAPK inhibitor)	Attenuate p38 phosphorylation	Attenuate mechanical allodynia	Xu et al.^77^
Lidocaine	Reduce expression of ERK 1/2 and CREB	Prevent and treat central neuropathic pain	Joo et al.^80^
JWH015 (cannabinoid type 2 receptor agonist)	Reduce p38 and ERK1/2 phosphorylation	Reduce neuropathic allodynia	Landry et al.^79^
Asiaticoside	Inhibit p38 MAPK pathway	Attenuate neuropathic pain	Luo et al.^78^
BMSCs	Suppress activation of p38 MAPK and ERK1/2	Relieve Pain Hypersensitivity	Watanabe et al.^81^
Rapamycin	Decrease p‐p38 MAPK‐positive microglia	Attenuate development of neuropathic pain	Tateda et al.^76^
Penehyclidine hydrochloride	Inhibit microglial MAPK/p‐p38/IL‐1b pathway	Attenuate neuropathic pain	Zheng et al.^75^
Regulators in inflammation			
Melatonin	Reduce activation of MAPKs p38, JNK and ERK1/2	Anti‐inflammatory effects	Esposito et al.^102^
C225 and AG1478 (EGFR inhibitor)	Inhibit EGFR/MAPK cascade	Reduce cytokine production in microglia	Qu et al.^93^
Tanshinone IIA	Inhibit activation of NF‐κB and MAPK signaling pathways	Decrease production of proinflammatory cytokines and reduced apoptosis	Yin et al.^100^
JQ1 (BRD4 inhibitor)	Suppress MAPK and NF‐κB signaling pathways	Suppress M1 polarization and proinflammatory cytokine production in microglia	Wang et al.^99^
Salidroside	Inhibit NF‐κB, p38 and ERK signaling pathways	Suppress expression of inflammatory cytokines and improve hind limb motor function	Su et al.^98^
Ursodeoxycholic Acid	Suppress phosphorylation of ERK, JNK, and p38 signals	Inhibit inflammatory responses and promotes functional recovery	Ko et al.^101^
5‐Methoxytryptophan	Suppress p38‐MAPK signaling pathway and NLRP3/caspase‐1 expression	Attenuate activated microglia	Hong et al.^97^
Kaempferol	Suppress phosphorylation of p38 MAPK and JNK	Reduce microglia activation and oxidative stress level	Liu et al.^103^
Rea	Inhibit both NF‐κB and MAPK signaling pathways	Promote M2 polarization of microglia, attenuated neuronal apoptosis	Xiao et al.^92^
Formononetin	Decrease p‐EGFR and p‐p38	Inhibit microglial inflammatory response	Fu et al.^94^
Stem cells and co‐transplantation system			
Drug‐releasing polymer in NSCs	Inhibit MAPK pathway	Inhibit NSCs Death	Yu et al.^119^
Collagen Nanofibers Used in NSCs	Increase phosphorylation of Synapsin I and facilitate the interaction of p‐ERK1/2 and p‐Synapsin I	Facilitate presynaptic maturation	Yin et al.^118^
BMSCs	Upregulate expression of cyclin D1 and p38 MAPK	Promote cell proliferation and inhibit apoptosis	Lin et al.^111^
GSI In iPSCs	Promote phosphorylation of p38 MAPK	Axonal regrowth	Okubo et al.^112^
UCMSCs	Inhibit p38 MAPK pathway	Suppresess neuronal apoptosis	Tian et al.^110^
Wnt4‐modified NSCs	Activate both β‐catenin and MAPK/JNK pathways	Promote differentiation of NSCs into neurons	Li et al.^113^
Metal–organic framework materials in DPSCs	Activate MAPK signaling pathway	Promote neural differentiation	Zhou et al.^117^
ncRNAs			
MiR‐30a‐5p	Target NeuroD1 through MAPK/ERK signaling	Ameliorate SCI‐induced inflammatory responses and oxidative stress	Fu et al.^134^
miRNA‐138	Suppress phosphorylation of JNK and the p38	Reduce H_2_O_2_‐induced apoptosis in BV‐2 cells	Ren et al.^131^
MicroRNA‐325‐3p	Inhibit EGFR/MAPK signaling pathway	Inhibit microglial activation and the release of inflammatory cytokines	Yan et al.^130^
miR‐340‐5p	Suppress expression of p38	Ameliorate SCI‐induced neuroinflammation and apoptosis	Qian et al.^133^
MicroRNA‐24‐3p	Inhibit MK2	Repress inflammation of microglia cells	Zhao et al.^129^
MiR‐152	Promote the expression of p38 MAPK	Inhibit neuronal axonal regeneration and apoptosis	Zhang et al.^135^
CircPrkcsh/miR‐488	Regulate MEKK1/JNK/p38 MAPK signaling pathway	Promote microglia M1 polarization	Li et al.^126^

### 7.1 Relief of neuropathic pain after SCI

Neuropathic pain, a prevalent complication affecting more than half of SCI patients, significantly deteriorates their health status and quality of life.^65^ The International Spinal Cord Injury Pain classification identifies the most common types of post‐SCI pain as musculoskeletal, visceral, or neuropathic.^66^ The complexity of neuropathic pain mechanisms post‐SCI involves structural and functional changes not only in the spinal cord near the injury site but also in peripheral areas, the thalamus, and the cortex. Current pharmacological interventions for neuropathic pain are tiered into three lines of treatment: first‐line drugs include anticonvulsants like pregabalin or gabapentin, possibly combined with tricyclic antidepressants such as amitriptyline; second‐line drugs incorporate weak opioids or substitute tricyclic antidepressants with 5‐hydroxytryptophan‐norepinephrine reuptake inhibitors; strong opioids, their use being controversial, are reserved as third‐line treatments. Invasive therapies, due to limited clinical evidence, are rarely proven effective.^67^ However, the therapeutic mechanisms of upon‐drugs have not been well elucidated. Among these, the mechanisms of action of some drugs are achieved through the MAPK signaling pathway. Therapy‐related studies of the MAPK signaling pathway in neuropathic pain post‐SCI have been shown (Figure S2).

#### 7.1.1 Neuropathic pain and DRGs post‐SCI

After SCI, dorsal root ganglions (DRGs), the structure of the neuroconduction system, are often destroyed. Consequently, neural impulses cannot be transmitted to and processed by the CNS, leading directly to the loss of various senses below the segment of SCI, pain included. Concurrently, the disruption of the blood–brain barrier allows peripheral inflammatory cells and factors to infiltrate the CNS, altering its microenvironment. Unsurprisingly, the excitability of intact DRGs is greatly enhanced by immune infiltration from both peripheral and CNS. This, coupled with the central sensitization of the spinal nerve after SCI, results in overexcited responses to diverse sensory stimuli, including pain.

Pain transmission occurs via nociceptors, primary afferent sensory fibers whose cell bodies, along with other primary sensory neurons, reside within the DRGs. DRGs are highly complex structures situated on either side of the spinal cord, spanning the length of the spinal column. Each DRG rises from the spinal cord dorsal horn (SCDH) as an enlargement of the dorsal root.^68^ It houses the cell bodies of primary sensory neurons, projecting axons both peripherally to injury sites and centrally to the dorsal horn of the spinal cord. Hence, it is conceivable that the DRG is a major site of nociceptive processing in both adaptive and maladaptive pain states.

Various membrane proteins implicated in the pain pathways are found in DRGs and expressed in nociceptors, including voltage‐gated ion channels, glutamate receptors and transporters, and G‐coupled receptors. Additionally, DRG neurons express numerous cytokines and chemokines and their receptors, underscoring the pivotal role of DRGs in nociception following tissue injury.^69^ The TWIK‐related spinal cord K+ (TRESK), a factor in neuropathic pain response, constitutes the major background potassium current in DRGs. Following SCI, TRESK suppression occurs concomitantly with MAPK signaling pathway activation, a process mitigated by ERK antagonist (PD98059) and p38 antagonist (SB203580).^70^

DRGs also exhibit unique combinations of tetrodotoxin‐sensitive (TTX‐S) and tetrodotoxin‐resistant (TTX‐R) Na^+^ channels, modulating resting membrane potential, action potential threshold, and neuronal firing frequency. Peripheral nerve damage, inflammation, and metabolic disorders alter the expression and function of these Na^+^ channels, heightening neuronal excitability and pain. These channels are targets of intracellular signaling cascades (e.g., protein kinases—PKA, PKC, and MAPK) that regulate their trafficking, cell surface expression, and gating properties. Injury‐induced alterations in these signaling pathways have been linked to sensory neuron hyperexcitability and pain.^71^

#### 7.1.2 Neuropathic pain and glial cells post‐SCI

Glial cells, especially microglia in the CNS, are key to the chronicity of pain mechanisms, both at peripheral injury sites and within the spinal cord's dorsal horn. Microglia are resident macrophage‐like immune cells in the CNS and are thought to be crucial in nerve pain.^72^ Among the three types of glial cells in the CNS, although oligodendrocytes and astrocytes are closely related to neurons, microglia have received more attention due to the fact that nerve injury‐induced changes in microglia are more intense than in oligodendrocytes and astrocytes. Nerve injury induces massive proliferation of spinal cord microglia,^73^ which release proinflammatory cytokines (IL‐1β, IL‐6, and TNF‐α) and a range of chemokines (including, but not limited to, monocyte chemotactic protein‐1 (MCP‐1), CCL20, CCL5, CXCL1, CXCL9, and CXCL10). These molecules exacerbate neuronal injury responses, escalating nociceptive sensitivity and persistent pain.^74^

The pathogenesis of neuropathic pain following SCI is intricately linked to the activation of the MAPK family, particularly p38. Thus, interventions targeting the p38 MAPK signaling pathway have shown efficacy in mitigating neuropathic pain. In studies conducted by Zheng et al.^75^ and Tateda et al.,^76^ significant strides were made in understanding the pathogenesis of neuropathic pain following SCI and the potential therapeutic interventions targeting the p38 MAPK signaling pathway. Zheng et al. demonstrated that intraperitoneal injection of phencyclidine hydrochloride effectively suppressed the p‐p38/IL‐1β pathway in the spinal microglia of rats, resulting in the alleviation of neuropathic pain induced by nerve damage. Similarly, Tateda et al. treated SCI‐afflicted mice with rapamycin, leading to a remarkable decrease in phosphorylated p38 MAPK‐positive microglia and suppression of spinal cord microglia activation. This treatment not only improved motor function post‐SCI but also significantly attenuated mechanical and thermal hypersensitivity responses, thus mitigating the development of neuropathic pain. Moreover, Xu et al.^77^ successfully lessened mechanical abnormal pain induced by chronic constriction injury by applying the p38 inhibitor SB203580 in a rat model. Additionally, Luo et al.^78^ demonstrated the potential of asiaticoside in pain management by inhibiting oxidative damage, nitric oxide activity, proinflammatory cytokine production, and the p38 MAPK pathway. Collectively, these findings underscore the effectiveness of intervening in the p38 MAPK signaling pathway to alleviate neuropathic pain. Inhibition of p‐p38 in various pathways associated with the MAPK pathway has proven instrumental in alleviating neuropathic pain, thereby ameliorating the stressful conditions such as oxidative stress.

In addition to these findings, other MAPK family members (JNK, ERK1/2 and ERK5), as well as atypical MAPKs, are under investigation for treating neuropathic pain. Notably, the cannabinoid type 2 receptor agonist JWH015 was studied for its ability to reduce neuropathic pain in rats by decreasing MAPK phosphorylation and inducing spinal MAPK phosphatases 1 and 3, the primary regulators of MAPK activity.^79^ Joo et al.^80^ suggested that lidocaine treatment may serve as an effective method to prevent and treat central neuropathic pain post‐SCI, attributable to its role in decreasing the expression of ERK 1/2 and CREB in intracellular signaling pathways. Additionally, Watanabe et al.^81^ discovered that early transplantation of bone marrow‐derived mesenchymal stem cells (BMSCs) can inhibit microglial activation of p38 MAPK and ERK1/2, thus suppressing neuropathic pain signaling pathways.

### 7.2 Suppression of neuroinflammation in SCI

#### 7.2.1 Spinal cord cellular microenvironment changes

In the intricate cellular microenvironment of the injured spinal cord, five key cell types including neurons, astrocytes, microglia, oligodendrocytes, and ependymal cells play essential roles (Figure S3). After SCI, these cells undergo significant changes in morphology, number, phenotype, and function, contributing to the development of inflammatory reactions at the injury site.

Astrocytes, the most abundant glial cells in the spinal cord, are crucial for neurotrophic support, synaptogenesis, synaptic maturation, and maintaining multimodal function.^82^ When activated post‐SCI, astrocytes transform into reactive astrocytes, classified into A1 and A2 types. A1 astrocytes are implicated in inducing neuronal and oligodendrocyte death, while A2 astrocytes exhibit protective, anti‐inflammatory effects and aid in neural repair post‐ischemic SCI.^83^, ^84^ Additionally, astrocytes are integral to scar formation, a key factor in managing inflammation spread.^85^ Microglia, akin to macrophages, perform immunosurveillance in the CNS under physiological conditions.^86^ Oligodendrocytes, vital for myelin formation, maintain axonal functionality.^87^ Neural stem cells, residing in the ependymal layer of the spinal cord's central canal, are typically quiescent with low proliferative activity under normal conditions.^88^

After SCI occurs, the spinal cord microenvironment undergoes drastic changes. Ependymal cells, often studied as a collective due to the current inability to fully distinguish individual cell compositions, include spinal cord‐derived neural stem cells. These cells, characterized by the expression of Sox2, Nestin, and Vimentin proteins, remain dormant in a healthy CNS but can differentiate to replace deceased neurons and glial cells post‐SCI, aiding in maintaining neural pathway integrity and neurological function restoration.^88^ Post‐SCI, oligodendrocytes undergo degeneration, leading to demyelination. Microglia numbers plummet in the central region of the injury, while blood‐derived macrophages become predominant, playing a crucial role in the injured zone.^89^ However, microglia are relatively active in the marginal zones, mainly exhibiting a proinflammatory M1 phenotype, releasing inflammatory factors, oxygen radicals and cytotoxic substances. Conversely, M2 microglia contribute anti‐inflammatory effects and promote tissue repair and regeneration.^90^

Therefore, in the context of SCI, astrocytes and microglia are the primary cells driving inflammation. Recent studies have focused more on these two cell types within the MAPK signaling pathway for SCI repair. In particular, there has been increasing research on modulating microglia through the MAPK signaling pathway as a therapeutic approach for SCI. Consequently, this article will primarily concentrate on microglia.

#### 7.2.2 Inhibition of microglial proliferation and differentiation and inflammatory response

The MAPK signaling pathway has an important regulatory role in the activation of microglia and the release of inflammatory factors leading to the inflammatory response. Efficient regulation of this pathway is crucial for SCI recovery (Figure S4). Morganti et al.^91^ investigated the acute and persistent inflammatory responses of focal traumatic brain injury (TBI) on microglia. By knocking out p38α in mouse microglia, they observed a diminished characterized by reduced production of proinflammatory cytokines/chemokines and diminished recruitment of inflammatory monocytes into the brain. This intervention also prevented persistent microglial morphological activation. Notably, the study revealed that amelioration of the inflammatory response correlated with expedited recovery of microglia to their basal phenotype, highlighting the pivotal role of microglia p38α in neuroinflammation following trauma. Similarly, Xiao et al.^92^ demonstrated that Rehmannioside A treatment inhibited the release of proinflammatory mediators from microglia in vitro, promoting M2‐type polarization, which in turn protected cocultured neurons from apoptosis through inhibition of the NF‐κB and MAPK signaling pathways.

Furthermore, in the SCI model, the EGFR signaling pathway, extensively studied in oncology, exhibits crosstalk with the MAPK signaling pathway. Qu et al.^93^ decreased EGFR expression using continuous infusion of C225 or AG1478 (both EGFR inhibitors) in SCI rats, leading to reduced levels of phosphorylation of ERK and p38 MAPK, activation of microglia, and astrocytes, as well as IL‐1β and TNF‐α levels, which demonstrated that inhibition of the EGFR/MAPK signaling pathway attenuated the inflammatory response of microglia and the associated secondary damage after SCI in rats. Additionally, with the injection of formononetin in mice, EGFR/p38 MAPK phosphorylation was inhibited, and formononetin showed benefits in improving motor function, repairing tissue damage and inhibiting microglial inflammatory responses.^94^ Pathological changes can lead to persistent inflammation in the injured area or even induce systemic inflammation in severe cases. The primary cause of this is the alteration of inflammatory factors induced by the activation of inflammatory cells, such as IL‐2, TNF‐α, anti‐GM1 ganglioside antibodies, IL‐4, and IL‐10.^95^, ^96^ Hong et al.^97^ explored the use of 5‐methoxytryptophan in treating spinal cord trauma, observing a reduction in proinflammatory microglia activation and diminished production of inflammatory cytokines such as TNF‐α, IL‐38β, IL‐3, and IL‐1. This effect was achieved through negative regulation of the p38 MAPK signaling pathway and expression of NLRP1/cystatase‐6. Furthermore, Su et al.^98^ were the first to report that salidroside (SAD) inhibited NF‐κB, p38, and ERK signaling pathways by WB analysis. Further, in vivo studies showed that SAD improved hindlimb motor function, reduced tissue damage, and inhibited the expression of inflammatory cytokines IL‐1β, IL‐6, and TNF‐α. Wang et al.^99^ successfully blocked lipopolysaccharide (LPS)‐induced activation of the MAPK signaling pathway in microglia using JQ1, a bromodomain protein 4 (BRD4) inhibitor, thereby attenuating the inflammatory response. Moreover, the application of tanshinone IIA,^100^ ursodeoxycholic acid (UDCA),^101^ and melatonin^102^ in SCI, the upregulation of phosphorylation levels of p38, ERK, and JNK was attenuated significantly in injured spinal cord tissues, suggesting they could attenuate MAPK signaling pathway activation, reduce proinflammatory factor production post‐SCI. Interestingly, Liu et al.^103^ demonstrated that pretreating BV‐2 cells with kaempferol resulted in reduced production of reactive oxygen species (ROS) by inhibiting NADPH oxidase 4. This inhibition, subsequently suppressed the phosphorylation of p38 MAPK and JNK, hindering the nuclear translocation of NF‐κB p65 and the expression of proinflammatory factors.

In conclusion, microglia are central to the inflammatory response following SCI. The upregulation of MAPK signaling pathway‐related proteins and factors is closely associated with microglia activation and inflammatory factor release. By inhibiting the phosphorylation level of p38, ERK, JNK, and other MAPK family members, the activation of the MAPK cascade responses post‐SCI, microglia activation, and inflammatory factor release are reduced. This diminishes the inflammatory response caused by secondary SCI, thereby protecting neurons to a certain extent and laying a foundation for future neurological function recovery.

### 7.3 Stem cell therapy in SCI and the MAPK signaling pathway

SCI can lead to profound motor, sensory, and autonomic dysfunction. Transplantation of Schwann cells,^104^ neural stem or progenitor cells,^105^ olfactory sheath cells,^106^ oligodendrocyte precursor cells and mesenchymal stem cells has been investigated as potential treatments for SCI.^107^, ^108^ However, the mechanisms by which these cell types facilitate repair and functional improvement are not fully understood. The commonly identified mechanisms that promote repair include neuroprotection, immunomodulation, axonal regeneration, neuronal relay formation and myelin regeneration.^109^ The role of the MAPK signaling pathway in stem cell therapy for SCI has garnered attention in this context (Figure S5). Tian et al.^110^ conducted a randomized controlled experiment using umbilical cord mesenchymal stem cells (UCMSCs), finding that UCMSCs promoted neurological function recovery in rats post‐injury. This improvement was attributed to the inhibition of the p38 MAPK pathway and reduced apoptosis in spinal cord neurons. The study highlighted the potential of UCMSCs in modifying the MAPK pathway to improve SCI outcomes. Conversely, while inhibition of neuronal apoptosis is beneficial, activation of the MAPK signaling pathway and upregulation of MAPK‐related components can facilitate stem cell differentiation and axonal regeneration. Lin et al.^111^ reported that overexpression of the *Nice4* gene in rat BMSCs led to enhanced cell proliferation and differentiation, concomitant with upregulation of p38 MAPK. This suggests that *Nice4* overexpression may promote functional recovery in SCI rats by bolstering cell proliferation and reducing apoptosis. The interplay between the Notch and MAPK signaling pathways is also evident in SCI repair studies. Okubo et al.^112^ found that treatment with a γ‐secretase inhibitor (GSI) that inhibited Notch signaling applied 1 day before transplantation of human induced pluripotent stem cells (iPSCs) promoted the phosphorylation of p38 MAPK, leading to axonal regeneration and potential neuronal maturation. Similarly, Li et al.^113^ demonstrated that Wnt4 significantly promoted neural stem cells (NSCs) differentiation into neurons and repaired neural circuits after SCI by activating the β‐linker protein and MAPK/JNK pathways, inhibiting Notch signaling.

The optimal timeframe for neural stem cells/precursor cells (NS/PCs) transplantation in SCI treatment is considered to be the subacute phase post‐injury.^114^, ^115^ Ensuring the survival and functionality of the graft is a crucial aspect of stem cell transplantation. Among the various therapeutic strategies in the field of repair and regeneration, hydrogels and nanoparticle (NP)‐based drugs stand out as the most effective, widely studied, and clinically valuable. Acting as drug carriers, hydrogels and NPs can be loaded with various drugs and biological therapeutic factors, allowing for slow release within SCI lesions. These carriers enhance drug efficacy by exerting anti‐inflammatory, antioxidant, and nerve regeneration effects to promote the recovery of neurological function.^116^ To this end, Zhou et al.^117^ used ZIF‐8‐transfected dental pulp stem cells (DPSCs) embedded in a methacrylated gelatin (GelMA) hydrogel for SCI treatment. This approach enhanced axonal growth and protected DPSCs from apoptosis, with ZIF‐8 activating the MAPK signaling pathway to promote neural differentiation and angiogenesis in the damaged microenvironment.

The survival of NSCs in acute SCI treatment is challenged by rapid donor cell loss and neuroinflammation. This problem can probably be solved by inhibiting the activation of the MAPK signaling pathway. By developing porous scaffolds composed of collagen nanofibers, Yin et al.^118^ employed the critical role played by the phosphorylation of synaptic proteins by MAPK/ERK1/2 kinases in synapse formation between spiral neurons in vitro, which is important for the proliferation of NSCs and synapse formation in differentiated neurons. Furthermore, Yu et al.^119^ experimentally demonstrated that apoptosis in human NSCs was mainly due to SIN‐1 treatment, which triggered protein nitration and activated the p38 MAPK pathway. They devised a co‐transplantation regimen using poly (lactic‐co‐glycolic acid) (PLGA) membranes embedded with ONOO (−) scavengers, significantly enhancing human NSCs survival and offering a promising approach for increasing graft and host cell survival post‐SCI.

### 7.4 Roles of non‐coding RNAs in SCI repair

Approximately 75% of the human genome is transcribed into RNA,^120^ while a mere 3% is transcribed into protein‐coding mRNAs.^121^ Noncoding RNAs (ncRNAs) are classified into various categories based on their length, shape, and location. Prominent among these are microRNAs (miRNAs), long noncoding RNAs (lncRNAs), circular RNAs (circRNAs), and PIWI‐interacting RNAs (piRNAs) are the four main ncRNA types, each playing distinct roles within cells through varied regulatory mechanisms.^122^ PiRNAs, initially identified in *Drosophila* and typically ranging from 24 to 30 nucleotides in length, are predominantly located in germ cells. They bind to PIWI family proteins and play a key role in the epigenetic regulation of chromatin.^123^ While miRNAs, lncRNAs, and circRNAs have been identified as emerging therapeutic targets for SCI, research into piRNAs in this context is less developed.^124^, ^125^, ^126^ The intervention of ncRNAs, particularly through the MAPK signaling pathway, is a burgeoning area of research in SCI therapy (Figure S6).

MiRNAs, approximately 22 nucleotides long, bind to complementary sequences in their target mRNAs, leading to degradation by the RNA‐induced silencing complex (RISC).^127^ These evolutionarily conserved nucleotides are crucial regulators in various physiological and pathological processes, primarily through base pairing with the 3′ untranslated region (3′UTR) of mRNA.^128^ Zhao et al.^129^ discovered that in injured spinal cord tissues in rats, miR‐24‐3p expression was significantly reduced compared to sham‐operated rats, alongside an upregulation of MK2, a downstream kinase of p38 MAPK, linked to the secretion of inflammatory cytokines like IL‐6, TNF‐α, and IL‐1β. Additionally, post‐SCI enhancements in the protein expression of Iba‐1, TNF‐α, and IL‐1β indicated activated microglia and an inflammatory response. Further experiments demonstrated that miR‐24‐3p could inhibit microglial activation and the production of inflammatory cytokines post‐SCI by regulating MK2 expression. Yan et al.^130^ explored miR‐325‐3p and its involvement in the EGFR/MAPK signaling pathway through in vitro and in vivo studies using rats with SCI and LPS‐activated primary microglia. Their findings showed that miR‐325‐3p might attenuate secondary injury post‐SCI by inhibiting the EGFR/MAPK signaling pathway, microglial activation and inflammatory cytokine release, indicating its potential as a therapeutic target for SCI. MiR‐138, which is downregulated during acute SCI, unlike miR‐325‐3p and miR‐24‐3p, appears to have a protective effect on microglia. Ren et al.^131^ found that overexpressing miR‐138 in BV‐2 cells treated with varying concentrations of H_2_O_2_ significantly reduced apoptosis rates. WB results revealed a downregulation of JNK, p‐JNK, c‐Jun, p‐c‐Jun, p38 MAPK, and p‐p38 MAPK, suggesting miR‐138's ability to reduce H_2_O_2_‐induced apoptosis in BV‐2 cells, likely through the downregulation of MLK3 protein, a crucial factor in the JNK/MAPK pathway.

Secondary injury in SCI is further characterized by increasing oxidative stress and inflammatory responses, where ROS‐mediated oxidative damage predominantly results from an imbalance between ROS production and protective mechanisms.^132^ As oxidative stress is a hallmark of SCI, alleviating it might be an effective therapeutic intervention. Qian et al.^133^ showed that miR‐340‐5p decreased microglial neuroinflammation, oxidative stress, and apoptosis in SCI rats. Increased miR‐340‐5p expression inhibited transduction through the p38 MAPK pathway to ameliorate neuroinflammation, thereby promoting recovery of neurologic function after SCI. Similarly, Fu et al.^134^ investigated the expression of the tumor suppressor gene miR‐30a‐5p in microglia post‐SCI and found that its overexpression significantly reduced inflammatory cytokines while promoting the expression of oxygen‐scavenging genes. This effect was similar to that achieved by silencing the *Neurod1* gene, suggesting miR‐30a‐5p's regulatory role on inflammatory factors and oxidative stressors through the NeuroD1/MAPK/ERK pathway.

Studies have consistently shown that miRNAs are pivotal in the pathogenesis of neuropathic pain and neuroinflammation due to nerve injury. Zhang et al.^135^ experimentally demonstrated that miR‐152 significantly promoted the activated p38 MAPK, leading to the expression of numerous apoptosis‐related genes and involvement in inhibiting neuronal axon growth and promoting apoptosis throughout SCI. Thus, the aberrant expression and function of miR‐152 emerge as critical determinants in the pathogenesis of SCI, unraveling promising avenues for targeted therapeutic interventions aimed at mitigating neurodegeneration and fostering regeneration in SCI patients.

LncRNAs and circRNAs, exceeding 200 nucleotides in length, differ structurally, with lncRNAs being linear and circRNAs being circular. Both can be transcribed from various genomic regions, including exons, introns, intergenic areas, or 5′/3′‐untranslated regions. They can fold into complex secondary structures, facilitating interactions with DNA, RNA, and proteins through multiple mechanisms, thereby regulating gene expression.^136^

Alterations in the expression of many lncRNAs have been shown to be an important part of CNS disorders, including neuropathic pain, neurodegenerative diseases, and others. Zhou et al.^137^ analyzed the functions of differential expression (DE) mRNAs by gene ontology (GO) annotation and pathway enrichment and summarized the characteristics of DE lncRNAs. In addition, a series of hub genes were identified that play key roles in SCI. These early mRNA and lncRNA expression changes may play a major role in the development of secondary injury. The enriched pathways analyzed by the Kyoto Encyclopedia of Genes and Genomes (KEGG) involved the toll‐like receptor pathway, p53 pathway, MAPK pathway, and Janus kinase (JAK)‐signal transducer and activator of transcription (STAT) pathway. This result suggested that differential mRNAs, as well as lncRNAs, could regulate the inflammatory response, apoptosis, and other important processes through the MAPK signaling pathway after SCI.

Circular RNAs (circRNAs) have emerged as critical players in the treatment of SCI. Studies have shown their significant roles in modulating various cellular processes associated with SCI pathogenesis and recovery. Li et al.^126^ verified the role of circPrkcsh (a circRNA) in microglia after SCI by in vitro and in vivo experiments. circPrkcsh was highly expressed in SCI and microglia. Upregulation of this gene promotes M1 polarization in microglia, leading to increased expression of inflammatory factors such as TNF‐α, IL‐1β, and IL‐6. High expression of circPrkcsh leads to elevated levels of MEKK1 mRNA as well as protein expression and upregulation of phosphorylated expression of JNK/p38 proteins, which was further demonstrated by double knockout rescue experiments, indicating that circPrkcsh regulated the MEKK1/JNK/p38 MAPK pathway through miR‐488. Therefore, we have reason to believe that in subsequent therapeutic studies on SCI, implantation of circRNA at the site of injury or introduction of circRNA via vectors can improve the local immune microenvironment of the injury, thus gradually realizing the purpose of rehabilitation.

### 7.5 Therapeutic studies in other aspects of SCI of the MAPK signaling pathway

In the realm of SCI treatment, fostering nerve cell regeneration and axonal extension is crucial for enhancing nerve function. Miao et al.^138^ employed Huntington‐associated proteins to augment neurological function in SCI mice by stimulating neural axon regeneration in vivo and enhancing neurite elongation in neuronally differentiated neurons from NSCs in vitro. This process was mediated via the Trka‐MAPK/ERK signaling pathway. Additionally, neuronal synapse remodeling, vital for the development and plasticity of the nervous system, involves rapid extension and retraction, reliant on neurotrophic factors such as NGF to promote neural synapse extension during brain development.^139^ Mufti et al.^139^ proposed that thrombin could have opposite effects in differentiated cells by activating different signaling pathways: the PI3K/AKT signaling pathway induced neural protrusion retraction and the ERK1/2 signaling pathway and p38 MAPK signaling pathway induced neural protrusion extension in differentiated cells. Further experiments showed that thrombin, by amplifying and sustaining the activation of ERK1/2 and p38 MAPK pathways, enhanced NGF‐mediated protrusion extension, thereby supporting NGF's role in neuronal differentiation and synapse elongation. Hu et al.^140^ developed a growth factor (GF)‐based delivery system (called GF‐HP) consisting of basic fibroblast growth factor (bFGF), NGF and heparin‐poloxamer (HP) hydrogel, which released NGF and bFGF in damaged spinal cords and improved neuronal survival, axonal regeneration and so on through the ERK/MAPK signaling pathway and the activation of the PI3K/Akt signaling pathway. In an effort to improve the pharmacologic regimen for SCI patients, an injectable hydrogel containing taurine deoxycholic acid (TUDCA) has been assembled and utilized for its anti‐inflammatory effects in SCI patients. The gel alone inhibited p‐ERK and p‐JNK in the MAPK pathway and reduced TNF‐α and glial fibrillary acidic protein (GFAP), markers of inflammation at the site of injury and was superior to the effects of saline, TUDCA and CHA (TUDCA and glycol chitosan‐oxidized hyaluronate mixed at a ratio of 9:1) gel alone.^141^

Hormone therapies, including those involving estrogen, progesterone and human chorionic gonadotropin (HCG), have demonstrated efficacy in improving SCI outcomes.^142^ Cheng et al.^143^ explored the role of the newly discovered membrane estrogen receptor, G protein‐coupled estrogen receptor 1 (GPR30 or GPER1), considering the feasibility of GPR30 agonists as estrogen substitutes. They found that GPR3 activation modulated the PI3K/Akt and MAPK/ERK pathways, increased GPR2 and anti‐apoptotic proteins Bcl‐3 and BDNF, and decreased pro‐apoptotic factor Bax and cleaved cysteine asparagin‐1, thereby exerting neuroprotective effects.

Photobiomodulation (PBM) is being used as an alternative therapy because of its effectiveness in soft tissue injury, wound healing, etc. The technique involves low‐power (1–500 mW) nonthermal delivery of photons in the visible or near‐infrared spectrum (405–1000 nm), which elicit beneficial biological responses in cells and tissues that are anti‐inflammatory and neuroprotective. The anti‐inflammatory effects of PBM are largely dependent on its ability to activate ATP‐dependent K^+^ channels and p38 MAPK.^144^

Extracorporeal Shockwave Therapy (ESWT), a non‐invasive pain management treatment that improves muscle strength via shockwaves, has shown therapeutic potential in various conditions, including degenerative osteoporotic and neurologic spinal lesions, heterotopic ossification due to SCI, cervical spondylosis, scoliosis, sacroiliac arthritis, and others. In vitro studies revealed that ESWT induces fluctuations in redox response regulation and an increase in the MAPK signaling pathway, stimulating enhanced gene expression in the nucleus.^145^

Furthermore, acupuncture, a prominent research area in China for SCI treatment, utilizes traditional Chinese medical elongated needle stimulation to regulate the MAPK family in SCI. This technique aims to inhibit apoptosis in the injured area and promote nerve function repair and regeneration.^146^

## 8 CLINICAL TRIAL FOR SCI RELATED TO MAPK SIGNALING PATHWAY

SCI poses a significant challenge in terms of treatment due to its extensive trauma and complex nature. Recognizing this urgent medical need, numerous researchers and organizations have dedicated efforts to enhance neurological function recovery and alleviate neuropathic pain in SCI patients. A plethora of cross‐disciplinary concepts and therapies, including human‐computer interaction (HCI), have been integrated into clinical trials for SCI treatment. We searched “EU Clinical Trials Register” and “clinicaltrials.gov” and found that the number of clinical trials on SCI treatment is very enormous. After meticulous filtering and comparison, we finally summarized the post‐SCI clinical trials related to the MAPK signaling pathway in the past 5 years (Table S1).

The categorization of clinical trials on MAPK signaling pathways for the treatment of SCI mirrors that of basic research, as described earlier. Firstly, cell transplantation therapy, which has demonstrated remarkable therapeutic potential in preclinical studies, is now a focal point in clinical practice. Whether involving stem cell transplantation or other cell therapies, these approaches aim to regulate the injured microenvironment and facilitate cellular repair. Notably, the MAPK signaling pathway plays a crucial role in regulating the survival, proliferation, and function of transplanted cells, as observed in previous applications of this pathway in stem cell therapy post‐SCI.

Another broad category of MAPK signaling pathway‐related clinical trials for SCI is drug therapy. These drugs can be broadly categorized into two main groups: those promoting axonal regeneration in injured neurons and those alleviating neuropathic pain post‐SCI. For instance, elezanumab is an isotype monoclonal antibody to human immunoglobulin (Ig) G1 that selectively binds to rejection guidance molecule A (RGMa), an inhibitor of axonal growth and important in inhibiting neuronal regeneration and functional recovery after CNS injury. Currently, elezanumab is being used to treat SCI in a phase 2 study. Moreover, it has been shown that RGMan is able to intervene in the MAPK signaling pathway and thus achieve the corresponding function.^147^ Second, Riluzole was able to increase the level of p38 MAPK, as well as the level of BDNP, which is undoubtedly essential for neuronal survival and axonal regeneration.

In clinical trials of relief of pathologic pain from SCI, although pregabalin was able to relieve the neuropathic pain associated with SCI, prolonged injections of pregabalin are toxic to neurons and can lead to neuronal apoptosis.^148^ This should be emphasized in the subsequent application of clinical work. Furthermore, gabapentin is often used as an antiepileptic drug in clinical practice. Surprisingly, the specific mechanism of its ability to inhibit the p38 MAPK signaling pathway and thus alleviate neuropathic pain has also been specifically elucidated.^149^ In addition, glibenclamide, an antidiabetic medication, has been used in clinical trials across a range of diseases. Glibenclamide is an ion channel blocker and has been shown to reduce neuroinflammation. This is very important for the improvement of the local immune microenvironment after SCI.

In summary, MAPK signaling pathway‐related clinical trials for the treatment of SCI are in full swing worldwide, with cellular therapies emerging as a prominent area of focus. Drug therapies, although traditional, the cross‐disease application of drugs has renewed research vigor in the traditional exploration of drug therapies. Some drugs that have long been widely used in clinical practice are also playing unexpected roles in new disease fields. Effective and safe therapies for SCI and its associated complications still need to be studied in depth.

## 9 CONCLUSION AND PERSPECTIVE

SCI is recognized globally as a serious health concern. Primary injuries are devastating to the anatomical structure and physiological function of the spinal cord, while secondary injuries further exacerbate the pathological changes of SCI. This exacerbation is mediated by the activation of multiple signaling pathways, among which the MAPK signaling pathway is paramount. Crucial for cell growth and development, the MAPK pathway governs key processes including proliferation, differentiation, stress response, immune reaction, adaptation, and apoptosis through its cascades—ERK1/2, the p38 family, JNKs, and ERK5. Extensive research has established the MAPK signaling pathway as a vital regulator in the pathophysiological progression of SCI. Numerous studies have confirmed that the MAPK signaling pathway plays a crucial regulatory role in the pathophysiological process of SCI, regulating neural stem cell differentiation, provoking neuropathic pain, and modulating glial cell activity.

Building upon the preceding section and additional research, it becomes evident that nearly all treatments targeting a singular signaling pathway, including the MAPK signaling pathway, represent a double‐edged sword. While such treatments offer highly targeted interventions with clear mechanisms and expected outcomes, it is crucial to acknowledge the multifaceted nature of biological systems. Organisms operate through a network of interconnected signaling pathways, and intervening in one pathway invariably triggers cascading effects on others. Thus, it is imperative to ensure that the role of other signaling pathways in such treatment modalities remains controllable and poses minimal biohazard risks. Failure to achieve this balance may lead to severe side effects, underscoring the need for meticulous regulation and a comprehensive understanding of biological responses to targeted interventions.

Presently, intervention strategies for SCI, grounded in research, predominantly fall into three categories: (1) The inhibition of inflammatory factors and cytokines release from microglia via the MAPK pathway, thus mitigating neuropathic pain post‐SCI. (2) The suppression of microglial activation and their inflammatory factors release through the MAPK pathway, thereby reducing secondary injury and the associated inflammatory response at the injury site. (3) The prevention of stem cell apoptosis via the MAPK pathway, which promotes the differentiation of NSCs and transplanted stem cells into neurons, as well as encourages axonal regeneration and extension, thereby enhancing nerve regeneration and functional recovery following SCI.

Given the multifaceted regulatory roles of the MAPK signaling pathway in SCI pathology, pinpointing specific functions is challenging. There are still many issues that need to be addressed in the current research on the MAPK signaling pathway in SCI. Firstly, SCI encompasses both acute and chronic phases; thus, identifying the optimal intervention timing within the MAPK pathway is crucial for effective recovery. Secondly, the complex cascade responses within the MAPK pathway suggest interconnectedness among the cascades, warranting further studies to discern whether these connections are inhibitory or promotive. Additionally, considering SCI's involvement of multiple cell types, precise regulation of the corresponding cells is essential for effective treatment. Current studies predominantly focus on microglia and NSCs, with less attention given to astrocytes and aspects like axonal and vascular regeneration within the MAPK pathway. Navigating the MAPK pathway's role in these challenges presents both opportunities and challenges for future SCI treatments. The development and application of the MAPK signaling pathway in these problems will be a challenge and an opportunity for SCI treatment in the future. Moreover, most existing studies on the MAPK pathway in SCI remain at cellular and animal experimental levels. Future directions should include exploring the MAPK pathway's specific mechanisms in clinical settings, developing effective therapies and targeted drugs, and devising inhibitors or methods targeting the MAPK pathway with high specificity and minimal adverse effects. We need to develop an inhibitor or inhibitory method with high specificity and few adverse effects to target the MAPK signaling pathway. Although the MAPK signaling pathway shows great potential for the treatment of SCI, the following issues still need to be addressed: (1) Potential interactions and specific mechanisms between the MAPK pathway and other activated signaling pathways post‐SCI. (2) Drug and stem cell therapies targeting the MAPK signaling pathway require more efficient and stable delivery systems. Therefore, the practicality, effectiveness, and safety of targeting the MAPK pathway for SCI treatment require comprehensive evaluation through further clinical studies. In conclusion, the MAPK signaling pathway plays a pivotal role in the post‐injury phase of SCI, intervening at the pathophysiological level in both treatment and neurological recovery post‐SCI.

## AUTHOR CONTRIBUTIONS

Xuhui Zhou, Xin Zhou, and W.L. designed the manuscript. S.H., Y.Z., and H.S. wrote and edited the manuscript. S.H. and Y.Z. created the figures. Xuhui Zhou, Xin Zhou, and W.L. supervised the project and revised the manuscript. All authors have read and approved the final manuscript.

## FUNDING INFORMATION

This work was funded by National Natural Science Foundation of China (82302677), Shanghai Sailing Program (23YF1458000) and Shanghai Chenguang Program (22CGA43).

## CONFLICT OF INTEREST STATEMENT

The authors declare that they have no competing interests.

## Supporting information


Data S1.

## Data Availability

Data sharing is not applicable to this article as no new data were created or analyzed in this study.
