# Supplementary material for: Advances of the MAPK pathway in the treatment of spinal cord injury
Source: CNS Neurosci Ther. 2024 Jun 17;30(6):e14807. doi: 10.1111/cns.14807 (PMC11183187; doi:10.1111/cns.14807)
Supplement: Supplementary file 1 — Data S1. [file CNS-30-e14807-s001.pdf]

Supplementary Figures and Figure legends

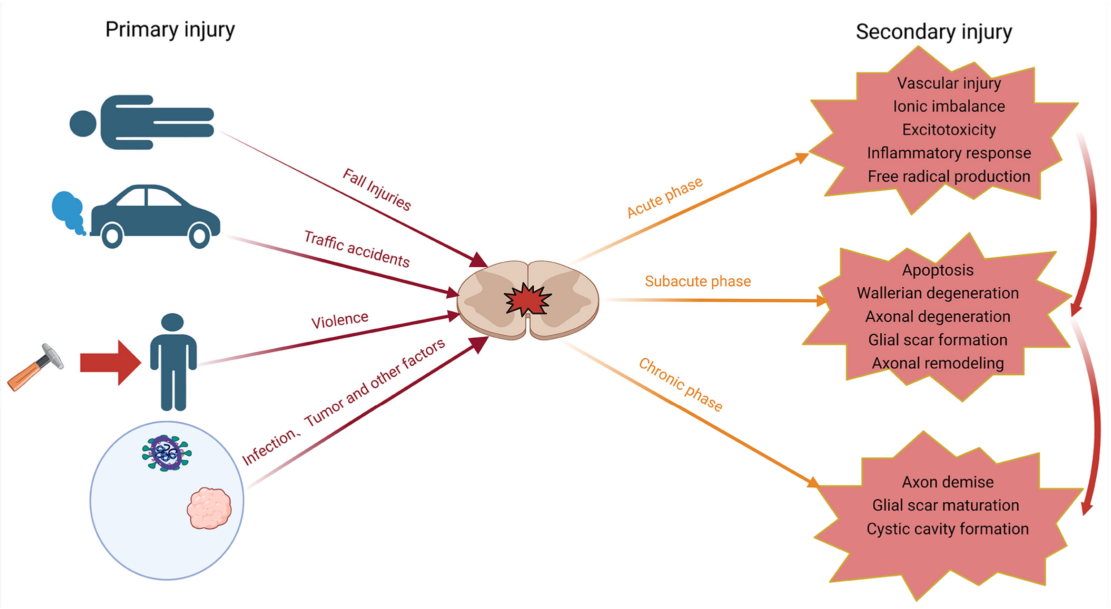

Fig. S1 Pathophysiological process following SCI. This diagram illustrates the progression post-SCI, initiated by primary damage from incidents like falls, traffic accidents, violence, infections and tumors. This leads to secondary damage encompassing acute, subacute and chronic phases, characterized by phenomena such as apoptosis, regeneration and inflammatory responses.

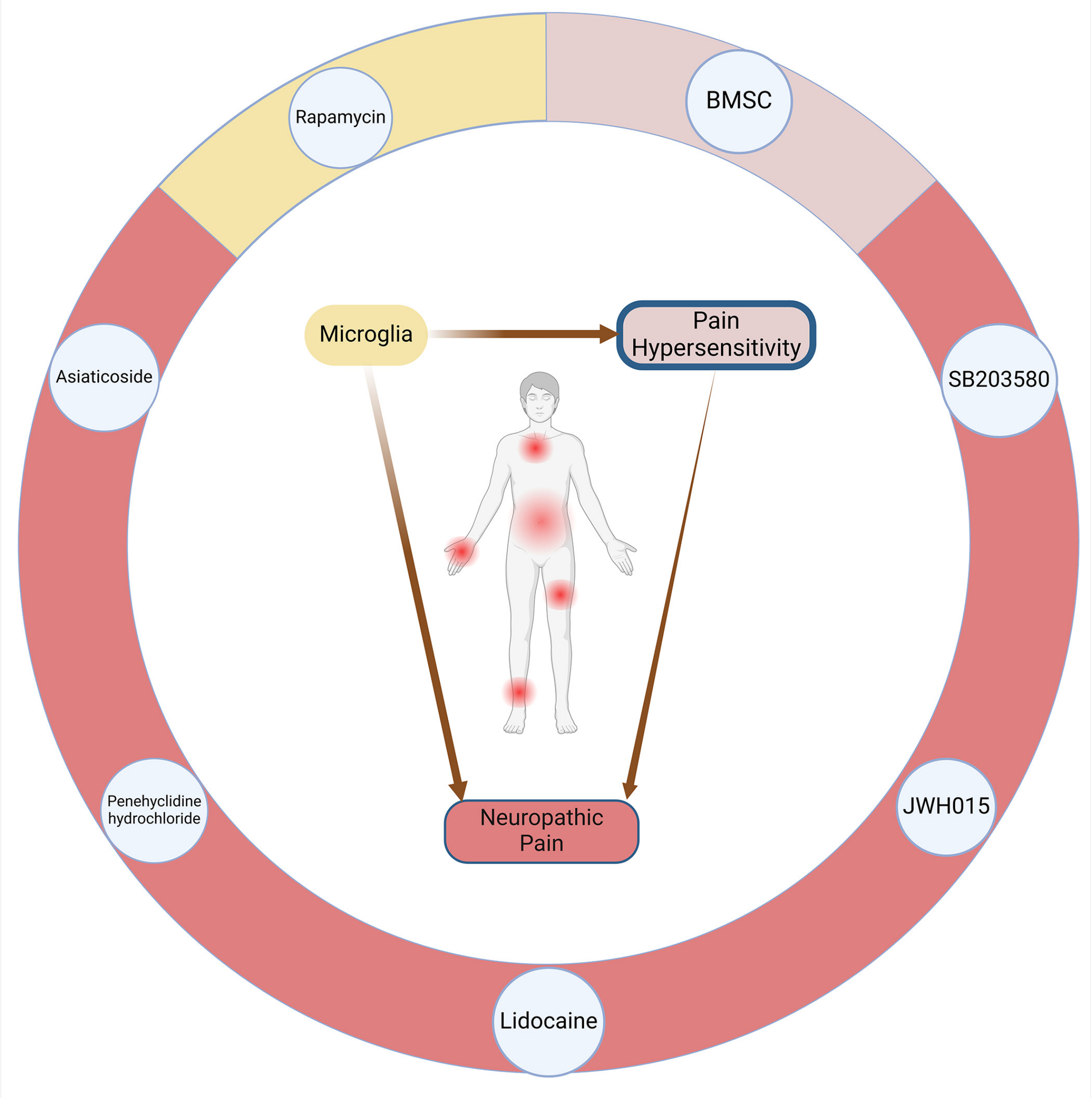

Fig. S2 Molecules in Neuropathic Pain Post-SCI. Highlights the significance of several molecules in the neuropathic pain relief cycle following SCI. The diagram focuses on three pivotal elements: microglia regulation, microglia control and pain hypersensitivity management.

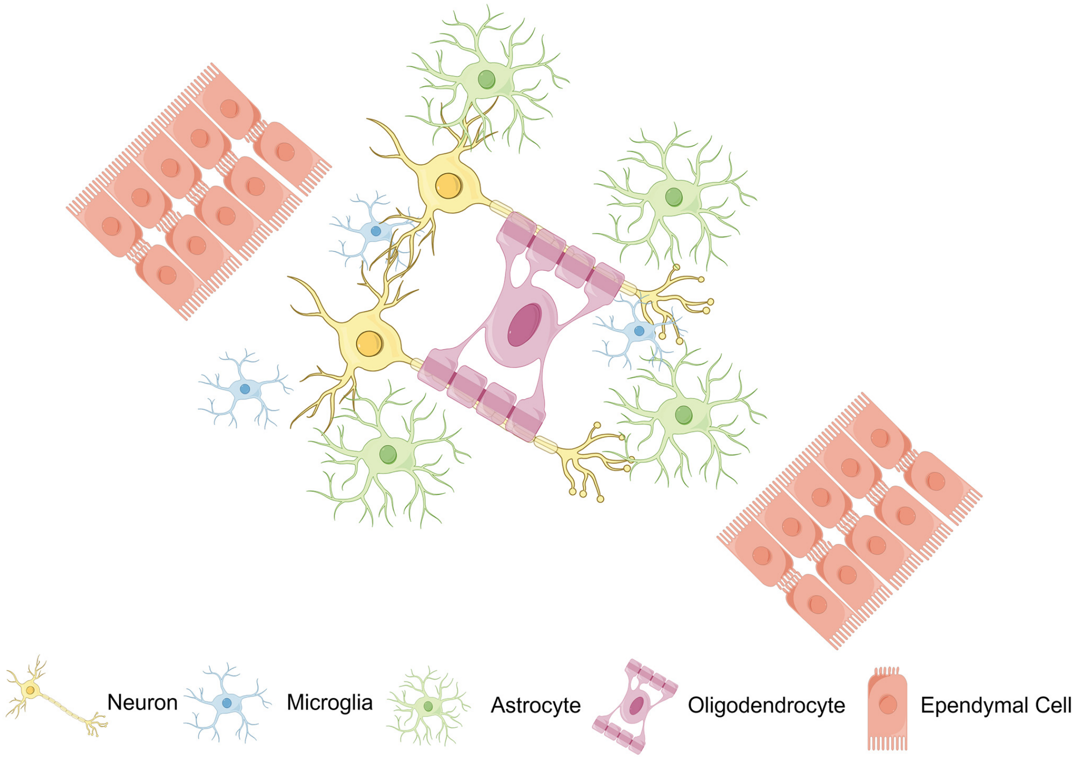

Fig. S3 Normal Human CNS Cells Functions. Illustrates the roles of various cells in a healthy CNS. Astrocytes, which regulate neurotransmitter secretion and ionic homeostasis; microglia, performing immunosurveillance; oligodendrocytes and their precursors, enhancing axonal conduction; and neural stem cells, predominantly quiescent in the central canal's ependyma ventriculorum. Post-SCI, there are several biological processes including direct and indirect neuronal death, microglia and astrocytes activation contributing to inflammation and glial scar formation, oligodendrocyte demise leading to demyelination and the differentiation of neuronal stem cells into glial cells and neurons for SCI repair.

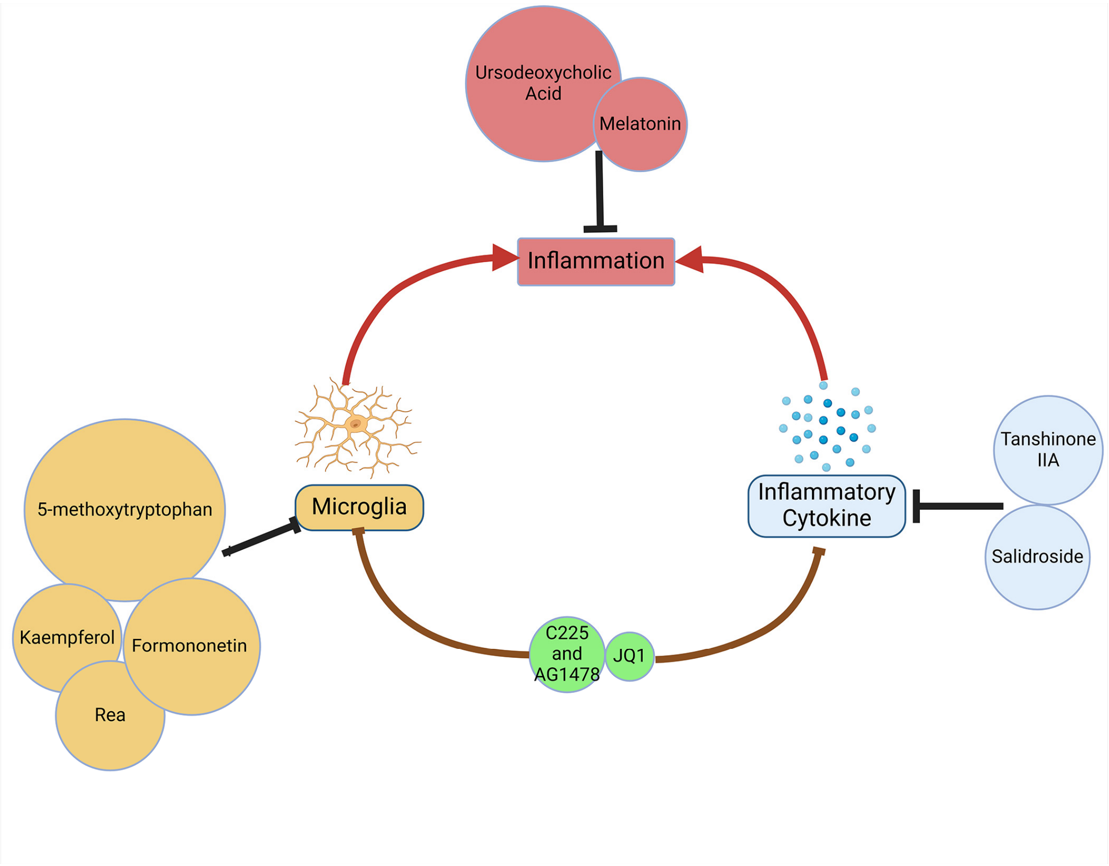

Fig. S4 Molecular Regulation of Inflammation Post-SCI. Emphasizes the crucial role of microglia and inflammatory cytokines in SCI-related inflammation, noting their direct impact on the cells and microenvironment at the injury site.

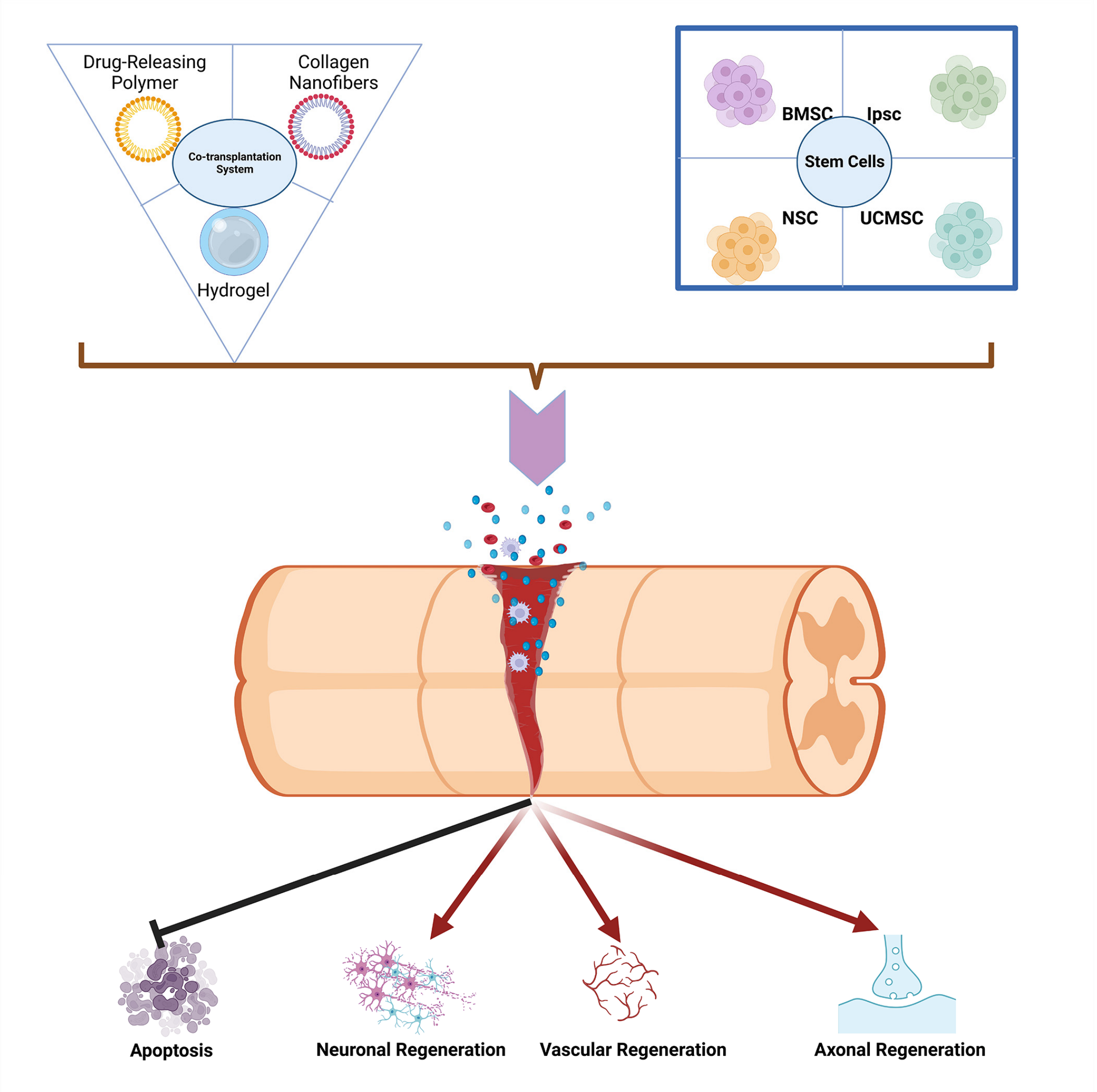

Fig. S5 Stem Cell Therapy for SCI via the MAPK Pathway. Presents the interaction between stem cell transplantation and co-transplantation systems in SCI treatment. The diagram explains how the co-transplantation system creates a supportive environment for stem cell transplantation, enhancing stem cell survival and subsequently promoting neuronal, axonal and vascular regeneration, aiding in the restoration of anatomical structures and neurological functions post-SCI.

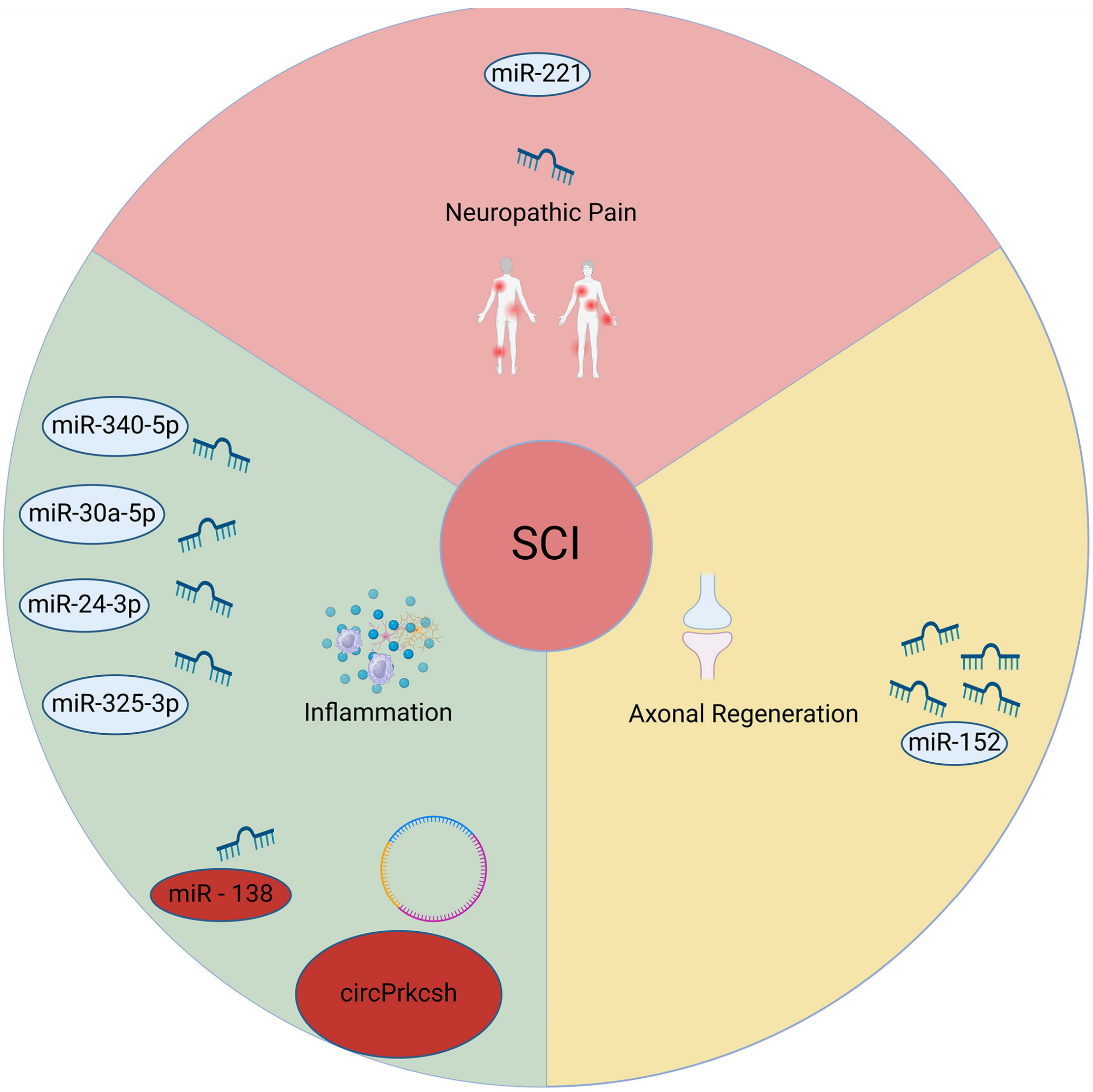

Fig. S6 Non-Coding RNA Regulation in SCI via the MAPK Pathway. Demonstrates the regulatory impact on healing in three main areas: neuropathic pain, neuroinflammation and axonal regeneration. The figure notes that while miR-138 and circPrkcsh can promote inflammatory responses, other ncRNAs typically inhibit inflammation, aid in axon regeneration and alleviate neuropathic pain.

Supplementary Table and Table legends

Table S1. Clinical Trials for SCI Related to MAPK Signaling Pathway

Clinical Trial Title	Sponsor Name	Start Date	Status	EudraCT Number/ClinicalTrials.gov ID	Stage
Riluzole in the Treatment of Spasticity in the Traumatic Chronic Spinal Cord Injury Condition	Assistance Publique Hopitaux De Marseille	2019.5.27	Complete	NCT02859792	Phase 2
Hyperbaric Oxygenation (HBO) in traumatic spinal cord injury - prospective study with 100 patients.	Division of Thoracic and Hyperbaric Surgery, Medical University Graz	2020.04.21	Ongoing	2018-004679-11	-
A Randomized, Double-Blind, Placebo-Controlled Proof of Concept Study to Assess the Safety and Efficacy of Elezanumab in Acute Traumatic Cervical Spinal Cord Injury	AbbVie Deutschland GmbH & Co. KG	2020.05.14	Ongoing	2019-003752-36	-

Safety And Efficacy Study of Intravenous (IV) Administration of Elezanumab to Assess Change in Upper Extremity Motor Score (UEMS) In Adult Participants with Acute Traumatic Cervical Spinal Cord Injury (SCI) (ELASCI)	AbbVie	2020.09.06	Ongoing	NCT04295538	Phase 2
Safety Stem Cells in Spinal Cord Injury (SSCiSCI)	Neuroplast	2020.11.14	Complete	NCT04205019	Phase1
Effects of Rimonabant on Walking Abilities in Incomplete Spinal Cord Injury: A Proof-of-concept Study	Hospital Nacional de Parapléjicos de Toledo	2021.5.12	Complete	NCT05398913	Phase 2
Prophylactic Pregabalin Treatment Following Spinal Cord Injury	Loewenstein Hospital	2021.7.13	Ongoing	NCT06066918	-

Cell Therapy for Chronic Traumatic Cervical Incomplete Spinal Cord Injury	Banc de Sang i Teixits	2021.10.13	Complete	NCT05054803	Phase 2
Feasibility of Early Gabapentin as an Intervention for Neurorecovery	MetroHealth Medical Center	2022.3.14	Ongoing	NCT05302999	Phase 3
Spinal Cord Injury Neuroprotection with Glyburide (SCING)	University of Kentucky	2022.7.7	Ongoing	NCT05426681	Phase1
Pharmacological Agents for Chronic Spinal Cord Injury (SCI)	Bronx VA Medical Center	2023.01.20	Ongoing	NCT05708274	Phase 1
